# A Novel Pre-Customized Saddle-Shape Soft Tissue Substitute for Volume Augmentation: An Ex Vivo Study in Pig Mandibles

**DOI:** 10.3390/ma18091951

**Published:** 2025-04-25

**Authors:** Malin Strasding, Irena Sailer, Elizabeth Merino-Higuera, Cristina Zarauz, Joao Pitta, Andrei Latyshev, Udo Wittmann, Dobrila Nesic

**Affiliations:** 1 Division of Fixed Prosthodontics and Biomaterials, University Clinic of Dental Medicine, University of Geneva, Rue Michel-Servet 1, CH-1211 Geneva, Switzerland; malin.strasding@unige.ch (M.S.); irena.sailer@unige.ch (I.S.); merinoprostodoncia@gmail.com (E.M.-H.); cristina.zarauz@unige.ch (C.Z.); joao.pitta@unige.ch (J.P.); andrei.latyshev@unige.ch (A.L.); 2 Center of Dental Medicine, Division of Periodontology and Peri-Implant Diseases, Clinic of Conservative and Preventive Dentistry, University of Zurich, Plattenstrasse 11, CH-8032 Zurich, Switzerland; 3 Dental Cabinet, Chapalita Avenue # 1255, Guadalajara 44500, Jalisco, Mexico; 4 Consult AG Statistical Services, Thurgauerstrasse 36, CH-8050 Zurich, Switzerland; udo.wittmann@consultag.ch

**Keywords:** soft tissue volume augmentation, soft tissue substitutes, Fibro-Gide, subepithelial connective tissue graft, customized graft, personalized dental medicine

## Abstract

Background: Tooth loss results in hard- and soft-tissue volume loss over time. We compared the handling of three different soft tissue substitutes (STS) to the subepithelial connective tissue graft (SCTG) for soft tissue volume augmentation in a pig ex vivo model. Methods: Five dentists simultaneously shaped, placed and sutured randomized four graft types in single-tooth soft tissue defects created in pig mandibles. The STS, produced from slightly crosslinked collagen fibres (VCMX), were either 3 mm or 6 mm thick blocks or a newly developed pre-customized saddle-shape. Each graft type was handled 20 times. The time required for shaping, placement, and suturing was recorded. Dentists reported outcomes on the grafts’ handling were evaluated with a visual-analogue-scale (VAS). Statistical analysis included calculating means and medians and testing significance. Results: The mean time of 0.72 min for shaping the pre-customized saddle-shape STS was significantly lower than 1.31 min for SCTG, 1.73 min for 3 mm STS and 2.17 min for 6 mm STS. Placement/suturing time was similar for all grafts. The dentists mainly preferred the saddle-shape STS and the SCTG. Conclusions: The saddle-shape STS required less time for graft-shaping and, therefore, reduced the overall treatment time, suggesting a more efficient and less invasive workflow for soft tissue augmentation.

## 1. Introduction

Tooth loss undeniably leads to a decrease in hard- and soft-tissue volume over time [1]. Sufficient bone volume is necessary for correct implant placement, and the soft-tissue quality and quantity are crucial in maintaining long-term peri-implant health [2,3]. Decreased hard and soft tissue volumes causing biological and esthetic problems often demand functional and esthetic treatments prior to tooth- or implant-borne restorations. Different hard and soft tissue augmentation procedures for optimal implant placement have been described [4,5]. In particular, the importance of oral soft-tissue volume stability over time has been demonstrated for optimal functional and esthetic outcomes [6,7].

The autogenous sub-epithelial connective tissue graft (SCTG) still represents the gold standard for soft tissue volume augmentation [8,9,10]. However, the restricted availability, necessity of a secondary surgical (donor) site, prolonged surgical time, and patient discomfort [11,12,13] continue to motivate research on soft tissue substitutes (STS). Ideally, STS should demonstrate biocompatibility, ease of shape adaptation to the defect site and subsequent placement, blood clot stabilization, volume maintenance, promotion of cellular migration/population, proliferation and differentiation, and finally, tissue integration. Based on these criteria, collagen-based matrices may be optimal for soft tissue regeneration [14,15]. Different types of STS are currently employed in periodontal and peri-implant volume augmentation procedures: allogenic, xenogeneic, synthetic and cell-containing living constructs [16,17,18,19]. Two xenogeneic STS, mainly used for soft tissue augmentation, are a porcine-derived acellular dermal matrix (Mucoderm, botiss biomaterials GmbH, Zossen, Germany) [20,21,22,23] and a volume stable matrix produced from reconstituted slightly cross-linked collagen fibres (Fibro-Gide, Geistlich Pharma AG, Wolhusen, Switzerland) [11,13,24,25,26,27,28,29,30]. However, both types of STS are delivered as prefabricated, standard-size blocks and must be customized according to the different 3D geometries of the individual defect sites, similar to SCTG. Whether the clinical handling of SCTG or of the prefabricated slightly crosslinked collagen STS blocks is more time-consuming remains controversial [25,26,27,28,29,30,31]. Additionally, the precision of trimming was shown to be dentist-dependent, resulting in a high variation in the STS-shape, especially concerning thickness. Even though the STS thickness exceeded the thickness of SCTGs, the observed buccal volume gain of the STS was more limited [25]. Due to the defect site exposure during the STS preparation time, the risk of infection is increased, thereby jeopardizing wound healing and the post-operative results. To reduce the chair-side adjustments of the prefabricated soft tissue blocks, STS should be produced in shapes that would better correspond to individual defects, and either be ready for insertion or require minimal chairside shape adjustments. Producing individualized STS still requires further development, yet fabrication of a pre-customized STS based on average defect shapes would allow easier handling, faster preparation and insertion of the STS, and if necessary, suturing.

Our previous studies have established and validated a standardized digital method to design an optimal STS shape for volume augmentation in single-posterior [32], double-posterior and single-anterior [33] soft tissue defects. We identified the seven most common shapes based on 71 defects. The present study aims to compare the shaping, placement and suturing of prefabricated standardized STS blocks and the newly designed “saddle-shape” pre-customized STS matrix, all based on reconstituted slightly cross-linked collagen fibres, to the SCTG in a pig mandible ex vivo model. The times required for grafts’ shaping and placement with flap suturing were recorded. The surgeon satisfaction, i.e., the Clinician Reported Outcomes (CROs) regarding the different grafts’ handling characteristics was also evaluated.

## 2. Materials and Methods

**Figure 1:**
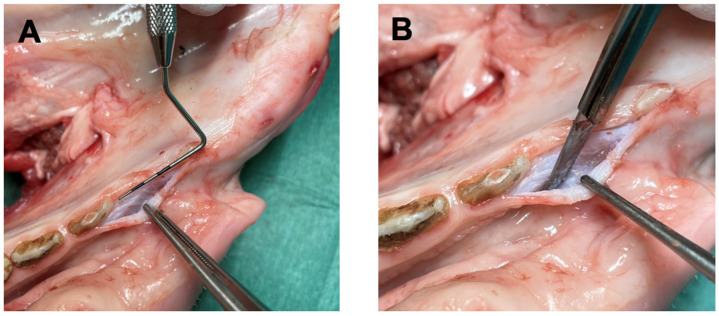
Photographs showing the preparation of a partial thickness flap encompassing the region from the mandibular first premolar to the canine. (**A**) Depiction of the size of the incision. (**B**) The use of scalpel to produce the defect.

Ethical approval was not required for this ex vivo study. The study was performed according to the CRIS Guidelines [34]. Forty fresh pig mandibles were acquired from the local slaughterhouse. A mid-crestal mucoperiosteal incision was performed bilaterally in the edentulous region between the canine and the first premolar to create a soft tissue defect on each side of the mandibles. The horizontal incision was extended to a sulcular incision of canine and first premolar. A partial thickness flap of 15 mm in length was raised without vertical incisions to remain minimally invasive (Figure 1). To ascertain standardization, one dentist (MS) practised the preparation procedure several times before creating identical defects/flaps for the study.

**Figure 2:**
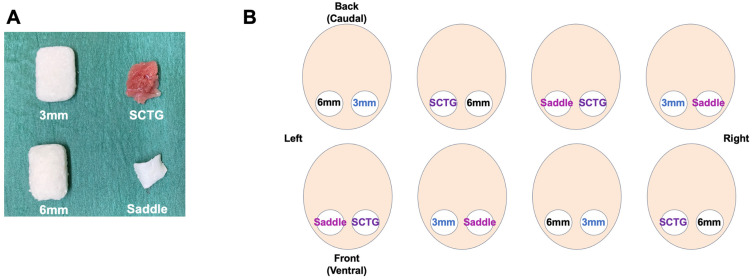
The four grafts treatment options and their positioning in pig mandible. (**A**) Three STS matrices (VCMX, GFG) of different shapes and sizes and the SCTG, harvested from other regions of the pig jaw. (**B**) Illustration of an example of the randomized arrangement option each dentist received for graft placement.

Five unbiased, experienced dentists performed the surgical procedures. For each dentist, an assistant was assigned to record time for graft shaping, and for placement and suturing, as well as to ensure the accuracy in proceedings. Each dentist/assistant team obtained a 6-well plate with four samples of each STS prototype (Geistlich Fibro-Gide^®^ (GFG or VCMX), Geistlich Pharma AG, Wolhusen, Switzerland): a 12 × 20 × 6 mm block (6 mm GFG), a 12 × 20 × 3 mm block (3 mm GFG), and a pre-customized “saddle-shape” (saddle GFG) with a thickness of 2.5 mm in the central area, gradually decreasing to the edges up to 0.5 mm and a length of 10 mm. The saddle GFG was digitally designed based on average single-tooth posterior defect identified in our previous study [32]. Only 20 saddle-shape prototypes could be produced, which limited the number of each prototype to 20 samples per group. These three STS groups were compared to the gold standard, the subepithelial connective tissue graft (SCTG), the size of 14 mm length, 10–12 mm width, and 2–3 mm thickness harvested from other regions of the pig jaw (Figure 2A). The defect positions were randomized, with the order of treatment options varying from one dentist to another (Figure 2B).

**Figure 3:**
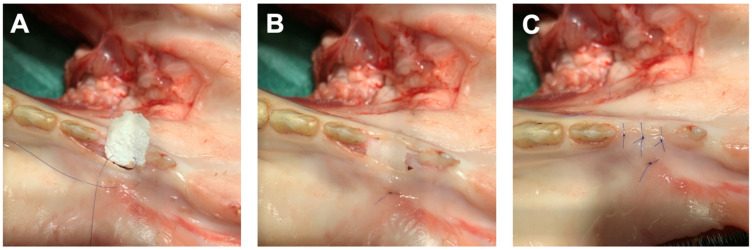
Photographs showing placement, suturing and closure of one STS graft. (**A**) Insertion of the graft using one apically placed suture to stabilize the graft’s position. (**B**) Final position of the graft at the site. (**C**) Tensionless primary flap closure with three single-knot sutures.

Each dentist received a micro-surgical set with the instruments (HU-FRIEDY Group, Chicago, IL, USA) for handling, shaping and suturing, and a container with Phosphate-Buffered Saline (PBS) for wetting the STS. Five dentists used each prototype four times which resulted in a total of 20 shaping, placement and suturing per group. The dentists had the freedom to use scissors, scalpel and tweezers to trim and adjust the grafts to obtain the desired shape prior to final placement in the defect and suturing. The grafts were inserted using one apically positioned suture (Dafilon 5/0, B. Braun Medical AG, Sempach, Switzerland) going through the flap, then through the graft and back through the flap again (Figure 3A). This approach allowed graft position stabilization (Figure 3B). The flap was closed tensionless with a monofilament suturing material (Dafilon 5/0, B. Braun Medical AG, Sempach, Switzerland) (Figure 3C).

**Figure 4:**
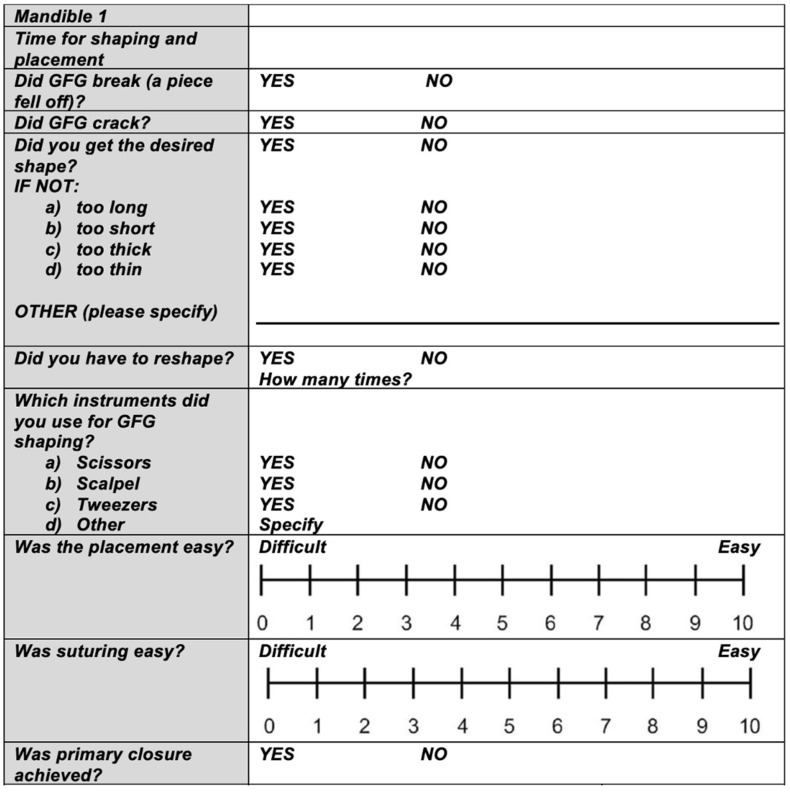
An example of the CROs recording sheet (questionnaire) given to the dentists to collect information regarding the time for shaping and the time for placement and suturing as well as handling of the different STS during the surgical procedures.

A recording sheet with specific questions was developed to document the surgical procedure (Figure 4). Primary outcome included time for STS shaping and time for placement. Secondary outcomes included the cracking/breaking of the STS; the necessity for reshaping and the type of instruments used were noted. A total of 16 sheets were completed per dentist. Additionally, the surgeons were asked to rank their preferences regarding the grafts’ handling from the easiest to the most difficult and provide their reasoning.

Statistical analysis comprised the assessment of numerical and binary variables. For the numerical variables, the mean and median with the corresponding bootstrap confidence intervals were calculated based on the Hodges–Lehmann estimator [35]. Numerical variables “number of reshaping times” and the analysis of the VAS score (1 to 10 values) for “easy placement” and “easy suturing” were first compared over the four groups with a Kruskal–Wallis test. In the case of significance, a pairwise comparison based on the median was performed with a Wilcoxon-rank sum test and a Bonferroni correction for multiple testing. For numerical variables “time for shaping” and “time for placement and suturing”, the logrank test was performed for pairwise comparison with the Bonferroni correction for multiple testing. Confidence intervals of the median were based on the cumulative hazard. Binary variables were expressed as percentages with confidence intervals. All data analysis were performed with the statistical package R, version 4.1.3 (R Foundation for Statistical Computing, Vienna, Austria, URL https://www.R-project.org/).

## 3. Results

**Figure 5:**
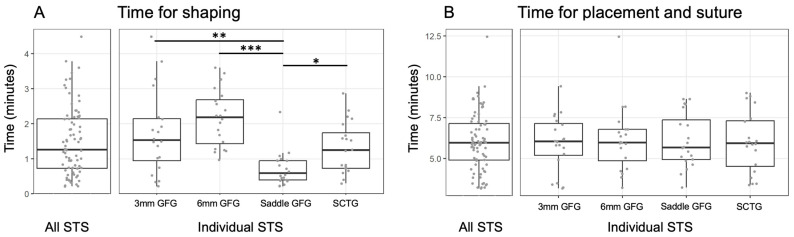
Graphical presentation of the analysis of times recorded by the dentist s. (**A**) Evaluation of the “time for shaping” and (**B**) “time for placement and suturing” for the four treatment grafts. Medians and confidence intervals are shown. Each graft was used 20 times (4 grafts per dentist). Significant differences (*p* < 0.05) are indicated: * for *p* < 0.05, ** for *p* < 0.01 and *** for *p* < 0.001.

The analysis of results for “time for shaping” showed a statistically significant difference between a pre-customized, saddle-shape, two block-shaped STS and the SCTG (20 samples per group). Shape adjustment of the saddle-shape required a median of 0.59 (CI 0.45. 0.95) (mean of 0.72; (0.5, 0.95)) minutes and was statistically significantly lower compared to 1.25 (CI 0.78, 1.98) (mean of 1.31; (0.98, 1.66)) minutes for SCTG, 1.53 (CI 1.05, 2.18) (mean of (1.73; 1.18, 2.28)) minutes for 3 mm STS and 2.18 (CI 1.68, 2.80) (mean of 2.17 (1.79, 2.54)) minutes for 6 mm STS (Figure 5A). By contrast, “time for placement and suturing” did not differ among the four groups: 6.08; 5.30, 6.86 (median 5.68, CI 5.12, 8.15) minutes for saddle-shape, 6.00; 5.14, 6.86 (median 5.94, CI 5.52, 7.45) minutes for SCTG, 5.95; 5.16, 6.74 (median 6.04; CI 5.65, 7.30) minutes for 3 mm STS and 6.17; 5.22, 7.11 (median 5.98; CI 5.65, 6.78) minutes for 6 mm STS (Figure 5B).

**Figure 6:**
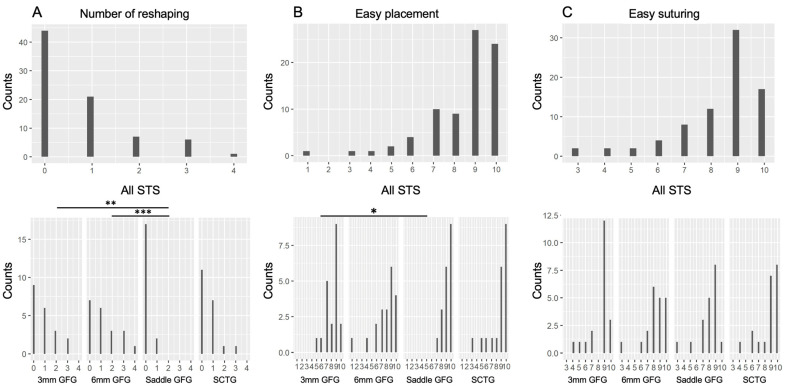
Graphical presentation of different STS handling parameters evaluated by the dentists. (**A**) Evaluation of the “number of reshaping times” and the VAS score (1 to 10 values) for (**B**) “easy placement”, and (**C**) “easy suturing”. Number of counts for the four treatment materials shown were calculated for the median with confidence intervals. n = 20. Significant differences (*p* < 0.05) are indicated: * for *p* < 0.05, ** for *p* < 0.01 and *** for *p* < 0.001.

The dentists evaluated several aspects of STS preparation and usage. The analysis of the number of times (counts) each graft had to be reshaped showed the lowest numbers for saddle type, a median of 0.0; CI 0.0, which were significantly lower compared to 3 mm block STS (median 1.0; CI 0.2) and 6 mm block STS (median 1.0; CI 0.1) but not to SCTG median 0.0; CI 0.1) (Figure 6A). The analysis of the CRO for easiness of placement based on the VAS score (counts) revealed that saddle type (median 9.0, CI 9.10) was significantly easier to place than 6 mm block STS (median 8.5; CI 7.9) but not 3 mm block STS (median 9.0; CI 7.9) or SCTG (median 9.0; CI 9.10) (Figure 6B). Concerning ease for suturing, also based on the VAS score (counts), 3 mm block STS appeared the easiest to suture, albeit statistically insignificant compared to other grafts (Figure 6C).

Several STS handling parameters were also evaluated. The desired shape was achieved in 90% of the cases (18/20) for SCTG, 3 mm block STS, and 6 mm block STS and in 75% of the cases (15/19) for saddle-shape. In 25% of saddle-shape cases, the desired shape was not achieved because the STS proved too short. In terms of STS cracking during the shaping process, 6 mm block STS presented cracks in 35% of the cases (7/20), 3 mm block STS in 25% of the cases (5/20) and saddle-shape in 20% of the cases (4/20). Regarding breaking, 3 mm block STS broke in 10% of the cases (2/20), saddle-shape in 5% (1/20), while 6 mm block STS did not break. Primary closure was achieved in all cases for all STS yet failed twice for SCTG. Among the instruments used to shape the grafts, a scalpel was mostly used (61%), followed by scissors (39%) and tweezers (19%). Some dentists also used fingers (20%).

Each surgeon had a different order of preferences for the STS. Saddle-shape and SCTG were found as the STS of choice twice each (40%), and 6 mm block STS was liked the least four times (80%).

## 4. Discussion

In this study, five experienced unbiased dentists evaluated the shaping, placement and suturing of four different soft tissue substitutes (STS) in an ex vivo pig mandible model of soft tissue volume defect. The obtained results indicate that the “saddle-shape” STS requires the least time for shaping and the least number of reshaping. The 6 mm block STS is the most difficult to place and the 3 mm block STS proves to be the easiest for suturing.

The duration and invasiveness of dental surgical procedures impact wound healing and patient-perceived pain [36,37]. Therefore, it remains important to reduce the intra-surgical treatment time as well as invasiveness reflected in a more extensive flap design. A recent RCT study reported no differences in the overall duration of the surgical procedures using either SCTG or the 6 mm block collagen-based STS (VCMX = GFG) [26]. However, no other VCMX shapes or sizes were compared. To follow on our previous in silico studies, where optimal average shapes were identified for single-tooth posterior [32] and anterior defects [33], the question remained whether a pre-shaped saddle type substitute would outperform the handling of the STS blocks in a pig mandible posterior single-tooth ex vivo model. Results showed that the pre-shaped saddle type substitute allowed faster (shorter time) and more efficient (fewer attempts of re-cutting) shaping. A reduced intra-surgical treatment time, which could be obtained with saddle type substitute, would benefit wound healing, treatment outcome and patient satisfaction.

In dental research, the pig-jaw model is a commonly used ex vivo model because the handling of the intraoral soft tissue resembles the one in humans [38,39,40]. Pig jaw models are also often used for teaching periodontal surgery procedures [41]. In this study, the pig mandible was chosen as the model because it allowed the evaluation of the STS placement and suturing under conditions similar to the human single-tooth edentulous sites. Although the created defect in a pig jaw is not identical to the single tooth-loss defect in humans, the handling and shaping time of the pre-shaped saddle type substitute is expected to show even better results compared to the 3 mm and 6 mm blocks STS or the SCTG when applied on human soft tissue defects. Time for placement and suture did not differ among the four treatment groups, further emphasizing that the initial step of graft shaping is critical for the overall treatment time.

The collagen matrices are designed to heal submerged; hence, the primary closure of a flap after insertion of an STS is critical. The saddle type substitute, designed to perfectly fit the defect by being thicker in the middle and thinner on the edges, would decrease the risk of wound dehiscence in comparison to a less well-defect-adapted one; namely, a block-shaped STS. Secondary wound healing caused by dehiscence would negatively impact the final outcome after soft tissue volume augmentation procedures. Thus, successful tensionless primary closure and primary wound healing are important to achieve a satisfactory, stable long-term result [31]. The potential exposure of the collagen matrix to the oral cavity would ensue remodelling and could lead to a loss of tissue volume upon resorption of the STS [25,26]. By contrast, SCTG does not depend on a submerged healing and a slight exposure of the SCTG and is therefore not problematic [26]. In the present study, primary flap closure was achieved for all collagen-based STS but not in two cases for SCTG. One study showed 67% of wound dehiscence after insertion of an 8 mm thick STS, in contrast to 90% in the case of SCTG [31]. 8 mm or the currently available 6 mm block STS used in several studies [26,28,29,42,43,44,45] largely exceeds the typical defect depth. Therefore, insufficient STS thickness reduction could result in excessive STS swelling upon contact with blood, leading to a wound dehiscence. In the ex vivo pig-jaw model, no blood circulation and saliva were present, leading to minimal STS swelling that could not be compared to a clinical setting. The primary closure is a parameter which should be interpreted with caution in the present ex vivo study.

In addition to the advantage of a reduced treatment time, an easier and less laborious shaping may improve CROs with the STS and the overall surgical procedure. In this study, each dentist had a different STS preference. The current gold standard, the SCTG and the novel saddle-shape substitute were preferred, while the thickest, 6 mm block STS, was the least liked. For the patients, however, according to PROMs, the use of STS is favoured over SCTG due to reduced pain perception [26]. To the authors’ knowledge, no studies report dentists’ satisfaction (CRO) with handling the different types of STS and SCTG.

Moreover, the saddle-shape substitute is thinly tapered at the edges in contrast to the even thickness of the 3 and 6 mm prefabricated STS blocks. The question of an over-augmentation potentially necessary to achieve an optimal result remains unclear. An extensive over-augmentation comes with a problematic primary closure, risk of tissue necrosis or increased remodelling, ultimately leading to tissue volume loss. Too little or no over-augmentation on the other hand may lead to a loss of volume gain in the long term. A certain amount of remodelling and subsequent volume loss has to be foreseen, yet there is currently no evidence of the exact extent of a potential volume over-augmentation requirement.

In addition to the issues pertaining to an ex vivo model, including lack of tissue elasticity and wet environment, another study limitation comprises the need for more familiarity of the dentists with the saddle type substitute prior to this very particular surgical procedure. Allowing the dentists previous handling of the different STS, particularly the saddle type substitute, could have reduced the steepness of the learning curve observed during the surgeries, namely the shorter times necessary for all STS shaping and suturing noted during the last procedures. Notably, the saddle type substitute was designed so that suturing for the graft stabilization on the defect could be omitted, as the well-adapted saddle-shape permits a stable position per se, without the need for the stabilizing sutures. To prevent bias, this was not communicated to the dentists, and they intuitively sutured all the grafts. Although a tempting possibility, whether the placement of the saddle type substitute without any fixation sutures is sufficient to maintain the graft in place during the healing period remains to be clinically verified.

In the future, the application of a pre-shaped STS for single-tooth soft-tissue defects may result in the production of better-fitted xenograft or synthetic STS grafts. One approach could be the production of saddle-shaped STSs of different dimensions for the treatment of a variety of soft tissue volume defects. For the individualized STS shapes design, however, the previously developed digital procedure [32,33] could be easily combined with the digital approach of intraoral scanning, thereby accelerating the soft tissue volume augmentation procedure. Moreover, for large defects comprising alveolar bone in addition to soft tissue, CBCT and intraoral scans could be combined to simultaneously augment hard- and soft-tissue volume according to the patients’ individual defect size and shape. Both approaches would reduce clinical time, enhance wound healing, reduce patient discomfort and potentially improve soft tissue volume augmentation.

## 5. Conclusions

The newly developed pre-customized, “saddle-shape” soft tissue substitute showed promising preliminary results in this ex vivo pig jaw study by demonstrating an overall reduced treatment time and thus a less invasive intervention when augmenting soft tissue volume in the single edentulous defects. The dentists’ satisfaction with handling the saddle-type STS was high. The application of a well-fitted graft to the individual defect shape would contribute to the further development of personalized dental medicine.

## Data Availability

The original contributions presented in this study are included in the article. Further inquiries can be directed to the corresponding author.

## Abbreviations

The following abbreviations are used in this manuscript:

SCTG	Subepithelial connective tissue graft
STS	Soft tissue substitute
GFG/VCMX	Geistlich Fibro-Gide
CRO	Clinician Reported Outcomes

## References

[1] Van der Weijden F., Dell’Acqua F., Slot D.E. (2009). Alveolar bone dimensional changes of post-extraction sockets in humans: A systematic review. J. Clin. Periodontol..

[2] Ackali A., Trullenque- Eriksson A., Sun C., Petrie A., Nibali L., Donos N. (2017). What is the effect of soft tissue thickness on crestal bone loss around dental implants? A systematic review. Clin. Oral Implant. Res..

[3] Gobbato L., Avila Ortiz G., Sohrabi K., Wang C., Karimbux N. (2013). The effect of keratinized mucosa width on peri-implant health: A systematic review. Int. J. Oral Maxillofac. Implant..

[4] Naenni N., Lim H.C., Papageorgiou S.N., Hämmerle C.H.F. (2019). Efficacy of lateral bone augmentation prior to implant placement: A systematic review and meta-analysis. J. Clin. Periodontol..

[5] Troeltzsch M., Troeltzsch M., Kauffmann P., Gruber R., Brockmeyer P., Moser N., Rau A., Schliephake H. (2016). Clinical efficacy of grafting materials in alveolar ridge augmentation: A systematic review. J. Cranio. Maxill. Surg..

[6] Avila-Ortiz G., Couso-Queiruga E., Pirc M., Chambrone L., Thoma D.S. (2023). Outcome measures and methods of assessment of soft-tissue augmentation interventions in the context of dental implant therapy: A systematic review of clinical studies published in the last 10 years. Clin. Oral Implant. Res..

[7] Del Amo F.S.L., Yu S.H., Sammartino G., Sculean A., Zucchelli G., Rasperini G., Felice P., Pagni G., Iorio-Siciliano V., Grusovin M.G. (2020). Peri-Implant Soft Tissue Management: Cairo Opinion Consensus Conference. Int. J. Environ. Res. Public Health.

[8] Ashurko I., Tarasenko S., Magdalyanova M., Bokareva S., Balyasin M., Galyas A., Khadimova M., Zhornik M., Unkoviskiy A. (2023). Comparative analysis of xenogeneic collagen matrix and autogenous subepithelial connective tissue graft to increase soft tissue volume around dental implants: A systematic review and meta-analysis. BMC Oral Health.

[9] Lissek M., Boeker M., Happe A. (2020). How Thick Is the Oral Mucosa around Implants after Augmentation with Different Materials: A Systematic Review of the Effectiveness of Substitute Matrices in Comparison to Connective Tissue Grafts. Int. J. Mol. Sci..

[10] Thoma D.S., Buranawat B., Hammerle C.H., Held U., Jung R.E. (2014). Efficacy of soft tissue augmentation around dental implants and in partially edentulous areas: A systematic review. J. Clin. Periodontol..

[11] Cairo F., Barbato L., Tonelli P., Batalocco G., Pagavino G., Nieri M. (2017). Xenogeneic collagen matrix versus connective tissue graft for buccal soft tissue augmentation at implant site. A randomized, controlled clinical trial. J. Clin. Periodontol..

[12] Lorenzo R., Garcia V., Orsini M., Martin C., Sanz M. (2012). Clinical efficacy of a xenogeneic collagen matrix in augmenting keratinized mucosa around implants: A randomized controlled prospective clinical trial. Clin. Oral Implants Res..

[13] Thoma D.S., Hilbe M., Bienz S.P., Sancho-Puchades M., Hammerle C.H., Jung R.E. (2016). Palatal wound healing using a xenogeneic collagen matrix—Histological outcomes of a randomized controlled clinical trial. J. Clin. Periodontol..

[14] Sculean A., Nikolidakis D., Nikou G., Ivanovic A., Chapple I.L., Stavropoulos A. (2015). Biomaterials for promoting periodontal regeneration in human intrabony defects: A systematic review. Periodontol. 2000.

[15] Wallace D.G., Rosenblatt J. (2003). Collagen gel systems for sustained delivery and tissue engineering. Adv. Drug Deliv. Rev..

[16] Tavelli L., McGuire M.K., Zucchelli G., Rasperini G., Feinberg S.E., Hang H.L., Giannobile W.V. (2020). Extracellular matrix-based scaffolding technologies for periodontal and peri-implant soft tissue regeneration. J. Periodontol..

[17] Toledano M., Toledano-Osorio M., Carrasco-Carmona A., Vallecillo C., Toledano R., Medina-Castillo A.L., Osorio R. (2020). State of the Art on Biomaterials for Soft Tissue Augmentation in the Oral Cavity. Part II: Synthetic Polymers-Based Biomaterials. Polymers.

[18] Toledano M., Toledano-Osorio M., Carrasco-Carmona A., Vallecillo C., Toledano R., Medina-Castillo A.L., Osorio R. (2020). State of the Art on Biomaterials for Soft Tissue Augmentation in the Oral Cavity. Part I: Natural Polymers-Based Biomaterials. Polymers.

[19] Wolff J., Farre-Guasch E., Sandor G.K., Gibbs S., Jager D.J., Forouzanfar T. (2016). Soft Tissue Augmentation Techniques and Materials Used in the Oral Cavity: An Overview. Implant Dent..

[20] Eeckhout C., Bouckaert E., Verleyen D., De Bruyckere T., Cosyn J. (2020). A 3-Year Prospective Study on a Porcine-Derived Acellular Collagen Matrix to Re-Establish Convexity at the Buccal Aspect of Single Implants in the Molar Area: A Volumetric Analysis. J. Clin. Med..

[21] Fickl S., Therese Kroger A., Dietrich T., Kebschull M. (2021). Influence of soft tissue augmentation procedures around dental implants on marginal bone level changes-A systematic review. Clin. Oral Implants Res..

[22] Papi P., Pompa G. (2018). The Use of a Novel Porcine Derived Acellular Dermal Matrix (Mucoderm) in Peri-Implant Soft Tissue Augmentation: Preliminary Results of a Prospective Pilot Cohort Study. Biomed. Res. Int..

[23] Puisys A., Deikuviene J., Vindasiute-Narbute E., Razukevicus D., Zvirblis T., Linkevicius T. (2022). Connective tissue graft vs porcine collagen matrix after immediate implant placement in esthetic area: A randomized clinical trial. Clin. Implant Dent. Relat. Res..

[24] Artzi Z., Renert U., Pirc M., Netanely E., Thoma D.S. (2022). Contour Changes Following Implant Placement and Concomitant Soft Tissue Augmentation Applying a Volume-Stable Collagen Matrix. Int. J. Periodontics Restor. Dent..

[25] Cosyn J., Eeckhout C., De Bruyckere T., Eghbali A., Vervaeke S., Younes F., Christiaens V. (2022). A multi-centre randomized controlled trial comparing connective tissue graft with collagen matrix to increase soft tissue thickness at the buccal aspect of single implants: 1-year results. J. Clin. Periodontol..

[26] Hammerle C.H.F., Jepsen K., Sailer I., Strasding M., Zeltner M., Cordaro L., di Torresanto V.M., Schwarz F., Zugh O., Akakpo D. (2023). Efficacy of a collagen matrix for soft tissue augmentation after implant placement compared to connective tissue grafts: A multicenter, noninferiority, randomized controlled trial. Clin. Oral Implant. Res..

[27] Naenni N., Bienz S.P., Benic G.I., Jung R.E., Hämmerle C.H., Thoma D.S. (2018). Volumetric and linear changes at dental implants following grafting with volume-stable three-dimensional collagen matrices or autogenous connective tissue grafts: 6-month data. Clin. Oral Investig..

[28] Thoma D.S., Gasser T.J.W., Hammerle C.H.F., Strauss F.J., Jung R.E. (2023). Soft tissue augmentation with a volume-stable collagen matrix or an autogenous connective tissue graft at implant sites: Five-year results of a randomized controlled trial post implant loading. J. Periodontol..

[29] Thoma D.S., Gasser T.J.W., Jung R.E., Hammerle C.H.F. (2020). Randomized controlled clinical trial comparing implant sites augmented with a volume-stable collagen matrix or an autogenous connective tissue graft: 3-year data after insertion of reconstructions. J. Clin. Periodontol..

[30] Zeltner M., Jung R.E., Hammerle C.H., Husler J., Thoma D.S. (2017). Randomized controlled clinical study comparing a volume-stable collagen matrix to autogenous connective tissue grafts for soft tissue augmentation at implant sites: Linear volumetric soft tissue changes up to 3 months. J. Clin. Periodontol..

[31] Thoma D.S., Zeltner M., Hilbe M., Hammerle C.H., Husler J., Jung R.E. (2016). Randomized controlled clinical study evaluating effectiveness and safety of a volume-stable collagen matrix compared to autogenous connective tissue grafts for soft tissue augmentation at implant sites. J. Clin. Periodontol..

[32] Sun Y., Yu T., Strasding M., Liu X., Burkhardt F., Schafer B., Sailer I., Nesic D. (2021). Design of customized soft tissue substitutes for posterior single-tooth defects: A proof-of-concept in vitro study. Clin. Oral Implants Res..

[33] Sun Y., Strasding M., Liu X., Schafer B., Liu F., Sailer I., Nesic D. (2023). Design of customized soft tissue substitutes for anterior single-tooth and posterior double-tooth defects: An in vitro study. J. Esthet. Restor. Dent..

[34] Krithikadatta J., Gopikrishna V., Datta M. (2014). CRIS Guidelines (Checklist for Reporting In-Vitro Studies): A concept note on the need for standardized guidelines for improving quality and transparency in reporting in-vitro studies in experimental dental research. J. Conserv. Dent..

[35] Hodges J.L., Lehmann E.L. (1963). Estimates of Location Based on Rank-Tests. Ann. Math. Stat..

[36] Gonzalez-Febles J., Romandini M., Laciar-Oudshoorn F., Noguerol F., Marruganti C., Bujaldon-Daza A., Zabalegui I., Sanz M. (2023). Tunnel vs. coronally advanced flap in combination with a connective tissue graft for the treatment of multiple gingival recessions: A multi-center randomized clinical trial. Clin. Oral Investig..

[37] Kofina V., Monfaredzadeh M., Rawal S.Y., Dentino A.R., Singh M., Tatakis D.N. (2023). Patient-reported outcomes following guided bone regeneration: Correlation with clinical parameters. J. Dent..

[38] Lau S.L., Chow L.K., Leung Y.Y. (2016). A Non-Invasive and Accurate Measurement of Gingival Thickness Using Cone-Beam Computerized Imaging for the Assessment of Planning Immediate Implant in the Esthetic Zone—A Pig Jaw Model. Implant. Dent..

[39] Mardas N., Dereka X., Donos N., Dard M. (2014). Experimental model for bone regeneration in oral and cranio-maxillo-facial surgery. J. Investig. Surg..

[40] Rocchietta I., Schupbach P., Ghezzi C., Maschera E., Simion M. (2012). Soft tissue integration of a porcine collagen membrane: An experimental study in pigs. Int. J. Periodontics Restor. Dent..

[41] Eswaran S., Dowlatshahi S., Weltman R., Zhu L., Elangovan S., Lee C.T. (2023). Preclinical teaching of periodontal surgical concepts using common instructional models: A comparative assessment. J. Dent. Educ..

[42] Hamdy A., Ibrahim S.S.A., Ghalwash D., Adel-Khattab D. (2024). Volumetric assessment of volume stable collagen matrix in maxillary single implant site development: A randomized controlled clinical trial. Clin. Implant Dent. Relat. Res..

[43] De Angelis P., De Angelis S., Passarelli P.C., Liguori M.G., Pompa G., Papi P., Manicone P.F., D’Addona A. (2021). Clinical comparison of a xenogeneic collagen matrix versus subepithelial autogenous connective tissue graft for augmentation of soft tissue around implants. Int. J. Oral Maxillofac. Surg..

[44] De Angelis P., Rella E., Manicone P.F., Liguori M.G., De Rosa G., Cavalcanti C., Galeazzi N., D’Adonna A. (2023). Xenogeneic collagen matrix versus connective tissue graft for soft tissue augmentation at immediately placed implants: A prospective clinical trial. Int. J. Oral Maxillofac. Surg..

[45] Schmitt C.M., Bruckbauer P., Schlegel K.A., Buchbender M., Adler W., Matta R.E. (2021). Volumetric soft tissue alterations in the early healing phase after peri- implant soft tissue contour augmentation with a porcine collagen matrix versus the autologous connective tissue graft: A controlled clinical trial. J. Clin. Periodontol..

